# Clues to Arsenic’s Toxicity: Microbiome Alterations in the Mouse Gut

**DOI:** 10.1289/ehp.122-A82

**Published:** 2014-03-01

**Authors:** Carol Potera

**Affiliations:** Carol Potera, based in Montana, has written for *EHP* since 1996. She also writes for *Microbe*, *Genetic Engineering News*, and the *American Journal of Nursing*.

Arsenic exposure has been linked to diabetes, cardiovascular disease, and cancers of the skin, bladder, lung, and liver.^1^ The mechanisms behind these human health effects are an ongoing area of research.^1^ The gut microbiome metabolizes arsenic, generating several intermediate compounds that are either more or less toxic than arsenic itself.^2^ In turn, ingested inorganic arsenic—the more toxic form of the metal—has been shown to change the composition of the gut community.^3^ Researchers exploring this newer angle report in *EHP* that arsenic exposure appears to alter not only the composition of the gut microbiome but also the metabolites it produces.^4^

Study leader Kun Lu of the University of Georgia, Athens, and colleagues exposed C57BL/6 mice to 10 ppm arsenic in drinking water for 4 weeks. Then they used 16S rRNA gene sequencing to compare the gut microbiome profiles of arsenic-exposed mice with those of untreated mice. Additionally, the team analyzed several hundred metabolites in blood, urine, and feces with liquid chromatography/mass spectroscopy to obtain a global portrait of how changes in the microbiome affected metabolic function.^4^

In control mice drinking arsenic-free water, the gut was populated predominantly with Bacteroidetes and Firmicutes families. Bacteroidetes populations remained similar in arsenic-treated mice, but several Firmicutes families significantly decreased. Firmicutes are important gut bacteria that produce short-chain fatty acids, which are used as substrates for energy production. Relatively high proportions of Firmicutes in the gut microbiota have been associated with obesity in humans.^5^^,^^6^

The investigators found that 146 metabolites increased and 224 decreased in arsenic-exposed mice, compared with unexposed mice. Among the altered metabolites were bile acids, lipids, amino acids, and isoflavones, some of which are linked to obesity, insulin resistance, and cardiovascular disease. For example, bile acids, which were significantly perturbed in arsenic-exposed mice, aid the absorption of lipids and fat-soluble vitamins from the gut.^7^ Bile acids also act as signaling molecules in lipid metabolism, and elevated levels have been associated with insulin resistance.^8^ “Bile acids may be potentially involved in arsenic-induced insulin resistance, but this needs to be confirmed,” says Lu.

**Figure:**
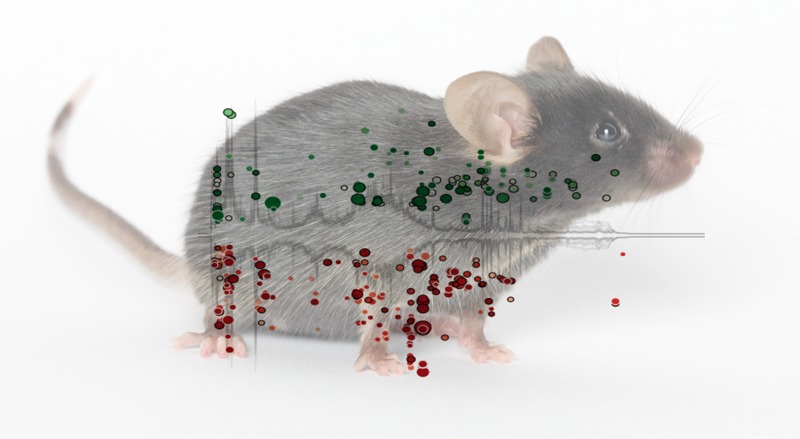
Arsenic exposure perturbed the metabolite profile of the mouse gut microbiome, increasing some metabolites (green) and decreasing others (red). Mouse: © The Jackson Laboratory. Scatterplot: Lu et al.; http://dx.doi.org/10.1289/ehp.1307429

Overall, the results provide preliminary clues for how environmental toxicants may contribute to human disease by disrupting the gut microbiome. In addition to arsenic, “we need to pay attention to the interactions of other environmental toxicants like mercury that also are metabolized in the gut,” says Lu.

Toxicologist Rebecca Fry of the University of North Carolina at Chapel Hill, who was not involved with the study, comments, “Given the likely probability that the effects [Lu and colleagues] observed in the mouse could occur in the human gut as well, the findings have great importance for public health as millions of individuals are exposed to harmful levels of arsenic in their drinking water.”

Worldwide, hundreds of millions of people drink water contaminated with inorganic arsenic levels that far exceed the 10-ppb guideline set by the U.S. Environmental Protection Agency.^9^ In the United States, arsenic is regulated in public drinking water, but an estimated 25 million people drink water from unregulated private wells with arsenic levels above 10 ppb.^10^

A logical next step would be to determine whether these findings in the mouse translate to humans and whether arsenic exposure is associated with changes in the human gut microbiome and metabolic profile. Lu says many other questions, including dose–response and gender effects and persistence of gut microbiome changes, need to be addressed in future animal studies.
